# The effects of thiamine pyrophosphate on ethanol induced optic nerve damage

**DOI:** 10.1186/s40360-019-0319-5

**Published:** 2019-07-05

**Authors:** Turgay Ucak, Yucel Karakurt, Gamze Tasli, Ferda Keskin Cimen, Erel Icel, Nezahat Kurt, Ibrahim Ahiskali, Halis Süleyman

**Affiliations:** 1 Department of Ophthalmology, Faculty of Medicine, Erzincan University, 24100 Erzincan, Turkey; 2 Department of Pathology, Faculty of Medicine, Erzincan University, Erzincan, Turkey; 3 Department of Biochemistry, College of Medicine, Atatürk University Hospital, Erzurum, Turkey; 4 Department of Ophthalmology, Erzurum Regional Training and Research Hospital, Erzurum, Turkey; 5 Department of Pharmacology, Faculty of Medicine, Erzincan University, Erzincan, Turkey

**Keywords:** Ethanol, Rats, Thiamine pyrophosphate, Oxidative stress, Glutathione, Malondialdehyde, Interleukin 1 beta, Tumor necrosis factor

## Abstract

**Background:**

We aimed to determine the protective effects of thiamine pyrophosphate on ethanol induced optic neuropathy in an experimental model.

**Methods:**

The rats were assigned into 4 groups, with 6 rats in each group as follows: healthy controls (HC group), only ethanol administered group (EtOH group), ethanol + thiamine pyrophosphate (20 mg/kg) administered group (TEt-20 group), and only thiamine pyrophosphate (20 mg/kg) (TPG group) administered group. To the rats in TEt-20 and TPG groups, 20 mg/kg thiamine pyrophosphate was administered via intraperitoneal route. To the rats in HC and EtOH groups, the same volume (0.5 ml) of distilled water as solvent was applied in the same manner. To the rats in TEt-20 and EtOH groups, one hour after application of thiamine pyrophosphate or distilled water, 32% ethanol with a dose of 5 g/kg was administered via oral gavage. This procedure was repeated once a day for 6 weeks. From the blood samples and tissues obtained from the rats, Malondialdehyde (MDA), reduced glutathione (GSH), interleukin 1 beta (IL-1β) and tumor necrosis factor alpha (TNF-α) levels were studied. Histopathological evaluations were performed to the optic nerve tissue.

**Results:**

Serum and tissue IL-1β, TNF-α and MDA levels were the highest in EtOH group which were significantly lower in thiamine pyrophosphate administered group (TEt-20 group) (p: 0.001). Serum and tissue reduced GSH levels were the lowest in EtOH group which were also significantly higher in TEt-20 group (p:0.001). In histopathological evaluations, in EtOH group there was obvious destruction and edema with hemorrhage and dilated blood vessels which were not present in any other groups.

**Conclusions:**

There was an apparent destruction in ethanol administered group in histopathological analyses with an augmented level of oxidative stress markers and all those alterations were prevented with concomitant thiamine pyrophosphate administration. These protective effects of thiamine pyrophosphate are extremely important in chronic ethanol consumption. Clinical studies are warranted to define the exact role of thiamine pyrophosphate in prevention of ethanol induced optic neuropathy.

## Background

Peripheral and optic neuropathies associated with chronic ethanol consumption are clearly known [1]. Although the etiology of ethanol induced optic neuropathy is multifactorial; malnutrition and direct toxic effects of ethanol are the main factors [2, 3]. Oxidative stresses, increased levels of free radical formation and elevated levels of pro-inflammatory cytokines has been shown in ethanol induced neuropathy [4–6].

Thiamine pyrophosphate is produced by phosphatisation of thiamine with thiamine pyrophosphokinase [7, 8]. It is the active metabolite of thiamine. Chronic alcohol consumption is known to decrease the expression of thiamine pyrophosphokinase enzyme; causing a decrease in thiamine pyrophosphate formation [9]. Previously, thiamine and thiamine pyrophosphate, both were studied in prevention of ethanol induced tissue damage on liver and only thiamine pyrophosphate was reported to be effective [10]. Moreover, recently, ethambutol induced oxidative stress has been shown histopathologically on the retinal tissue with thiamine pyrophosphate being effective in preventing this [11].

In this study, we aimed to determine the effects of thiamine pyrophosphate in prevention of etanol induced optic nerve damage in an experimental model. To the best of our knowledge, this is the first study in literature evaluating the biochemical and histopathological effects of thiamine pyrophosphate on ethanol induced optic nerve damage.

## Methods

The study was approved by the local ethics committee of Ataturk University, Erzurum, Turkey (Ethics Committee Number: 137, Date: 10.26.2017). All animal experiments were performed in accordance with the ARVO Statement for the Use of Animals in Ophthalmic and Vision Research.

### Study animals

Totally 24 albino Wistar male rats (265–278 g) were housed at room temperature (22 °C), fed twice a day and had access to water ad libitum *for 1 week before the study.* The rats were obtained from the Ataturk University Medical Experiments Application and Research center.

The rats were divided into 4 groups, with 6 rats in each group as follows: healthy controls (HC group), only ethanol administered group (EtOH group), ethanol + thiamine pyrophosphate (20 mg/kg) administered group (TEt-20 group), and only thiamine pyrophosphate (20 mg/kg) administered group (TPG group).

### Experimental procedure

In this experiment, to the rats in TEt-20 and TPG groups, 20 mg/kg thiamine pyrophosphate was administered via intraperitoneal route. To the rats in HC and EtOH groups, the same volume (0.5 ml) of distilled water as solvent was applied in the same manner. To the rats in TEt-20 and EtOH groups, one hour after application of thiamine pyrophosphate or distilled water, 32% ethanol with a dose of 5 g/kg was administered via oral gavage. Different doses of ethanol were used to produce oxidative stress in organs and tissues of animals. In our study this dose was preferred because 32% ethanol was more effective at a dose of 5 g /kg [12]. In literature, it was reported that alcohol results in oxidative damage in 6 weeks on optic nerve [13]. For that reason, this procedure was repeated once a day for 6 weeks with the same dose and volume in the same manner. At the end of this period, the animals were sacrificed with high dose (50 mg/kg) i.p. thiopental sodium (IE Ulagay-Türkiye) anesthesia and their eyes were enucleated.

### Biochemical evaluations

Blood samples were obtained from the tail veins of the rats and collected into separation gel vacutainer serum tubes. Blood samples were centrifuged at 1500 rpm for 15 min and stored at − 80 °C until biochemical analysis [14]. From these samples serum Malondialdehyde (MDA), reduced glutathione (GSH), interleukin 1 beta (IL-1β) and tumor necrosis factor alpha (TNF-α) levels were investigated.

Eye tissues of the rats were obtained after sacrification and the tissues were homogenized in ice-cold phosphate buffers and centrifuged at 5000 rpm for 20 min at 4 °C. Then IL-1β, TNFα, GSH and MDA levels were investigated from those supernatants. The protein concentration of the supernatant was also measured using the method described by Bradford MM and all tissue results were expressed by dividing to g protein [15].

#### IL-1β and TNF-α analysis in serum and tissue

Serum and tissue-homogenate IL-1β and TNF-α concentrations were measured using rat-specific sandwich enzyme-linked immunosorbent assay Rat Interleukin 1β ELISA Kit (Cat no: YHB0616Ra, Shanghai LZ) and Rat Tumor Necrosis Factor α ELISA kits (Cat no: YHB1098Ra, Shanghai LZ). The manufacturers’ instructions were followed for the analyses.

#### GSH analysis in serum and tissue

GSH levels in serum and tissue were analyzed regarding the method defined by Sedlak J and Lindsay RH [16]. For this purpose, the yellow color obtained through the reduction of DTNB (5, 5′-dithiobis [2-nitrobenzoic acid]) was analyzed by spectrophotometry at 412 nm.

#### MDA levels in serum and tissue

Based on the method used by Ohkawa et al. [17], MDA levels were measured by analyzing the spectrophotometrical absorbance of the pink-colored complex formed by thiobarbituric acid (TBA) and MDA at 532 nm.

### Histopathologic analyses

For histological examinations, eye tissues were fixed in 10% formalin for 24 h, and embedded in paraffin blocks. Four-micrometer-thick sections of specimens were de-paraffinized in xylene and dehydrated in graded alcohol [14]. The total retinal thickness, outer nuclear layer (ONL), inner nuclear layer (INL) and ganglion cell layer (GCL) were evaluated in H&E stained retina sections by the same pathologist.

### Statistical analysis

The statistical analyses were performed with the Software SPSS version 21.0 (SPSS for Windows software; SPSS Inc., Chicago, IL). Numerical variables were expressed as mean ± standard deviation. For the analysis of continuous variables one way variance analysis was performed. Subsequently, in determination of different groups Turkey multiple comparison tests were performed. Statistical significance was set at *p* < 0.05.

## Results

The results of biochemical analyses are summarized in Tables 1 and 2. Regarding these results, serum IL-1β, TNF-α and MDA levels were the highest in EtOH group which were significantly lower in thiamine pyrophosphate administered group. Similarly, tissue IL-1β, TNF-α and MDA levels, studied on optic nerve tissue, were also the highest in EtOH group which were significantly lower in thiamine pyrophosphate administered group. In TEt-20 group all those parameters were statistically significantly higher than both HC and TPG groups but significantly lower than EtOH group. On the other hand, serum and tissue reduced GSH levels were the lowest in EtOH group which were significantly higher in thiamine pyrophosphate administered group. In TEt-20 group those parameters were statistically significantly lower than both HC and TPG groups but significantly higher than EtOH group. There was not any statistically significant difference between HC and TPG groups regarding any of the study parameters.

**Table 1:** The results of biochemical evaluations of serum samples among groups

	HC (n:6)	EtOH (n:6)	TEt-20 (n:6)	TPG (n:6)	p
sIL-1β	3.60 ± 0.35^b,c^	8.15 ± 0.35^a,c,d^	4.23 ± 0.48^a,b,d^	3.83 ± 0.36^b,c^	**0.001**
sTNF-α	2.85 ± .018^b,c^	6.53 ± 0.41^a,c,d^	3.51 ± 0.31^a,b,d^	3.08 ± 0.194^b,c^	**0.001**
sMDA	1.75 ± 0.18^b,c^	4.78 ± 0.19^a,c,d^	2.21 ± 0.27^a,b,d^	1.58 ± 0.24^b,c^	**0.001**
sGSH	5.91 ± 0.26^b,c^	1.61 ± 0.33^a,c,d^	5.16 ± 0.16^a,b,d^	6.31 ± 0.33^b,c^	**0.001**

*HC* Healthy controls, *EtOH* ethanol group, *TEt-20* ethanol + thiamine pyrophosphate group, *TPG* thiamine pyrophosphate group. *MDA* Malondialdehyde, *GSH* total glutathione, *IL-1β* interleukin 1 beta, *TNF-α* tumor necrosis factor alpha, *s* serum. ^a^: statistically significantly different compared with HC; ^b^: statistically significantly different compared with EtOH; ^c^: statistically significantly different compared with TEt-20; ^d^: statistically significantly different compared with TPG. (Statistical significance was set at *p* < 0.05)

**Table 2:** The results of biochemical evaluations of tissue samples among groups

	HC (n:6)	EtOH (n:6)	TEt-20 (n:6)	TPG (n:6)	p
tIL-1β	2.18 ± 0.19^b,c^	4.98 ± 0.37^a,c,d^	2.71 ± 0.19^a,b,d^	2.51 ± 0.21^b,c^	**0.001**
tTNF-α	1.10 ± 0.12^b,c^	3.21 ± 0.23^a,c,d^	1.70 ± 0.14^a,b,d^	1.38 ± 0.13^b,c^	**0.001**
tMDA	0.95 ± 0.03^b,c^	3.31 ± 0.27^a,c,d^	1.35 ± 0.18^a,b,d^	0.92 ± 0.03^b,c^	**0.001**
tGSH	4.91 ± 0.24^b,c^	1.41 ± 0.36^a,c,d^	4.10 ± 0.31^a,b,d^	5.30 ± 0.34^b,c^	**0.001**

*HC* Healthy controls, *EtOH* ethanol group, *TEt-20* ethanol + thiamine pyrophosphate group, *TPG* thiamine pyrophosphate group. *MDA* Malondialdehyde, *GSH* total glutathione, *IL-1β* interleukin 1 beta, *TNF-α* tumor necrosis factor alpha, *t* tissue. *HC* Healthy controls, *EtOH* ethanol group, *TEt-20* ethanol + thiamine pyrophosphate group, *TPG* thiamine pyrophosphate group. *MDA* Malondialdehyde, *GSH* total glutathione, *IL-1β* interleukin 1 beta, *TNF-α* tumor necrosis factor alpha, *s* serum. ^a^: statistically significantly different compared with HC; ^b^: statistically significantly different compared with EtOH; ^c^: statistically significantly different compared with TEt-20; ^d^: statistically significantly different compared with TPG. (Statistical significance was set at *p* < 0.05)

**Fig. 1:**
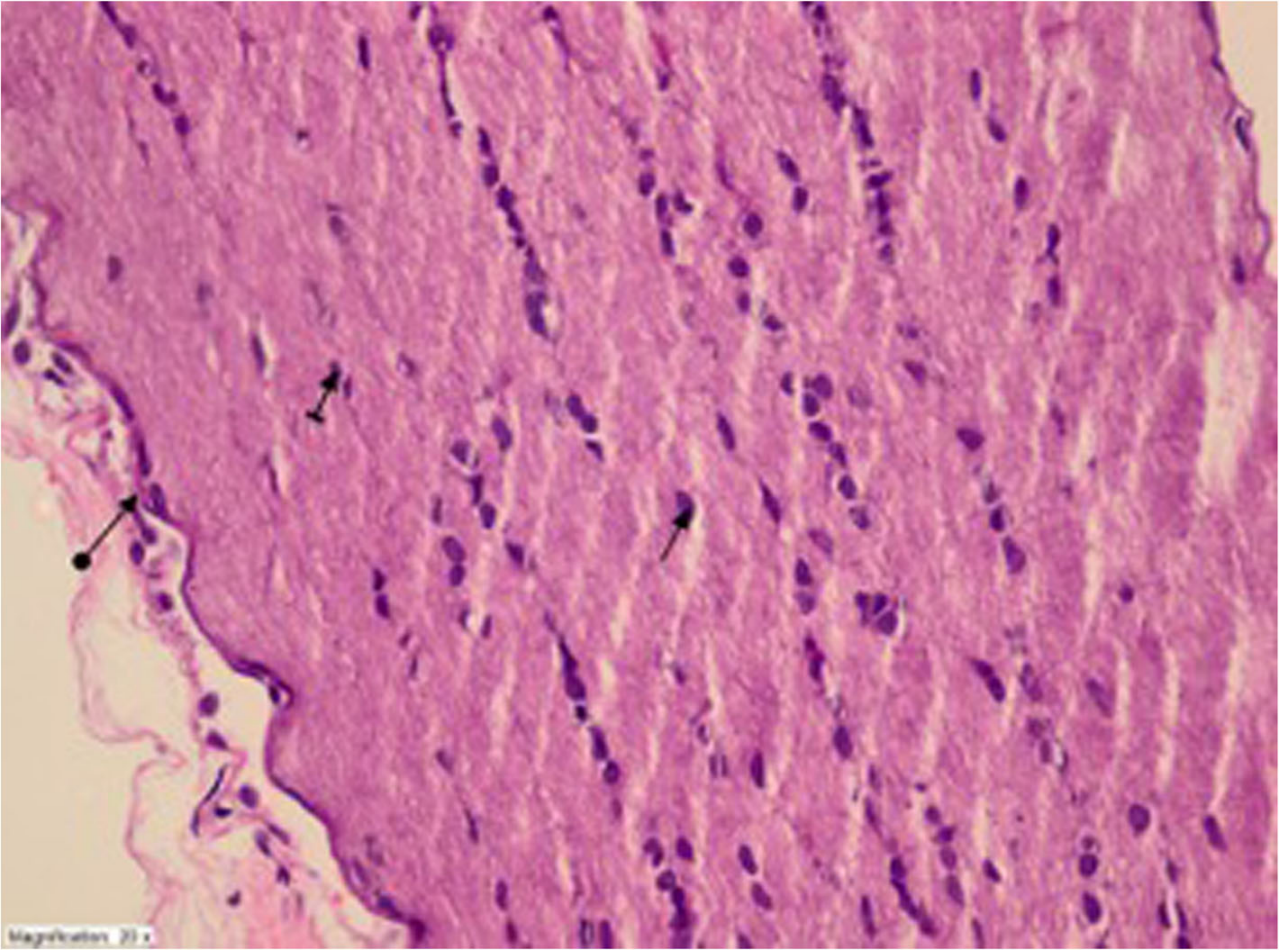
Optic nerve tissue of healthy controls- healthy nerve sheet (round arrow), healthy astrocyte (straight arrow), and healthy oligodendrocyte (striped arrow) (HEX400)

**Fig. 2:**
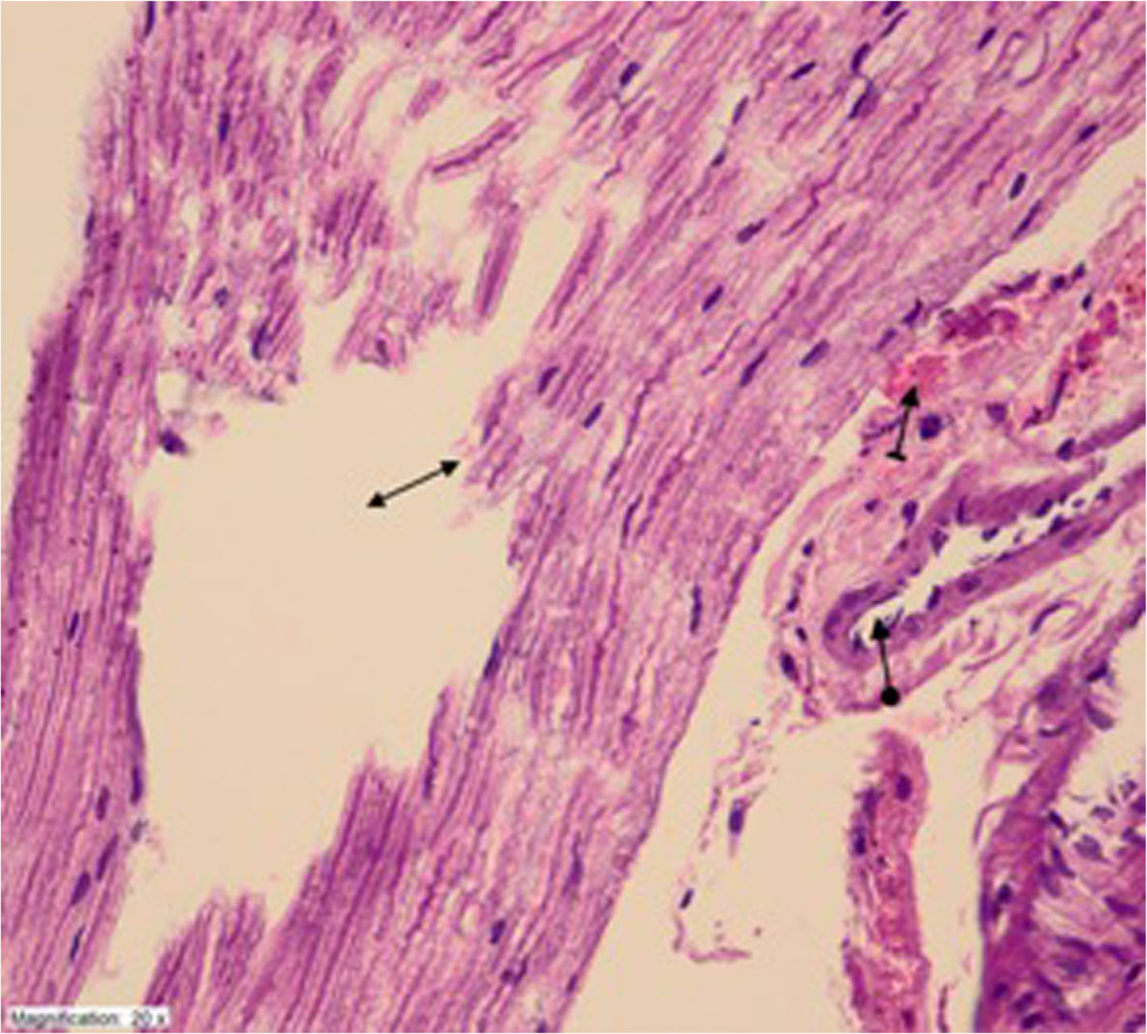
Obvious destruction and edema (double sided arrow) with hemorrhage (striped arrow) and dilated blood vessels (circular arrow) in ethanol administered EtOH group (HEX400)

**Fig. 3:**
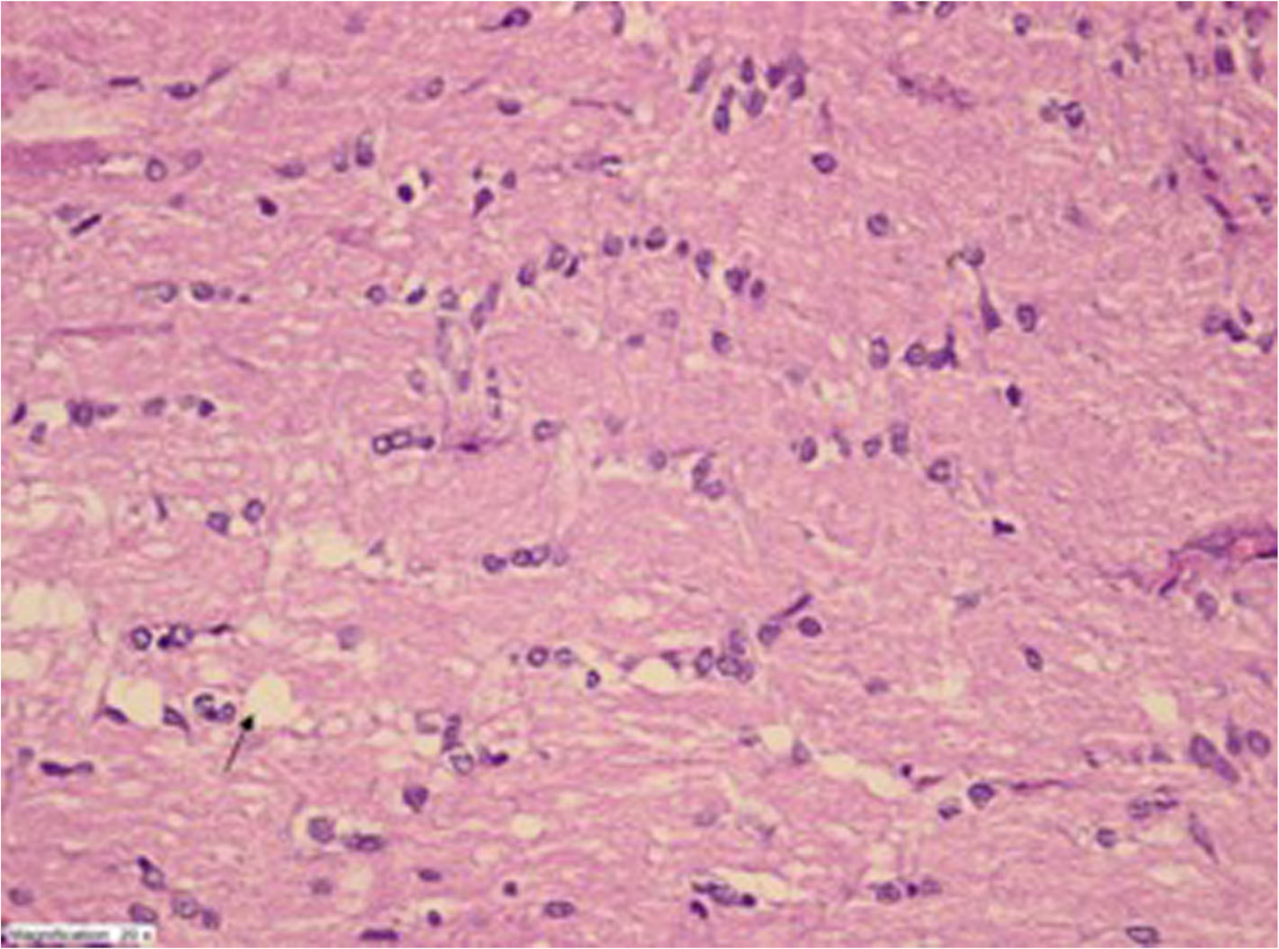
Mild degeneration and edema but otherwise normal appearance in optic nerve tissue of TEt-20 group (HEX400)

**Fig. 4:**
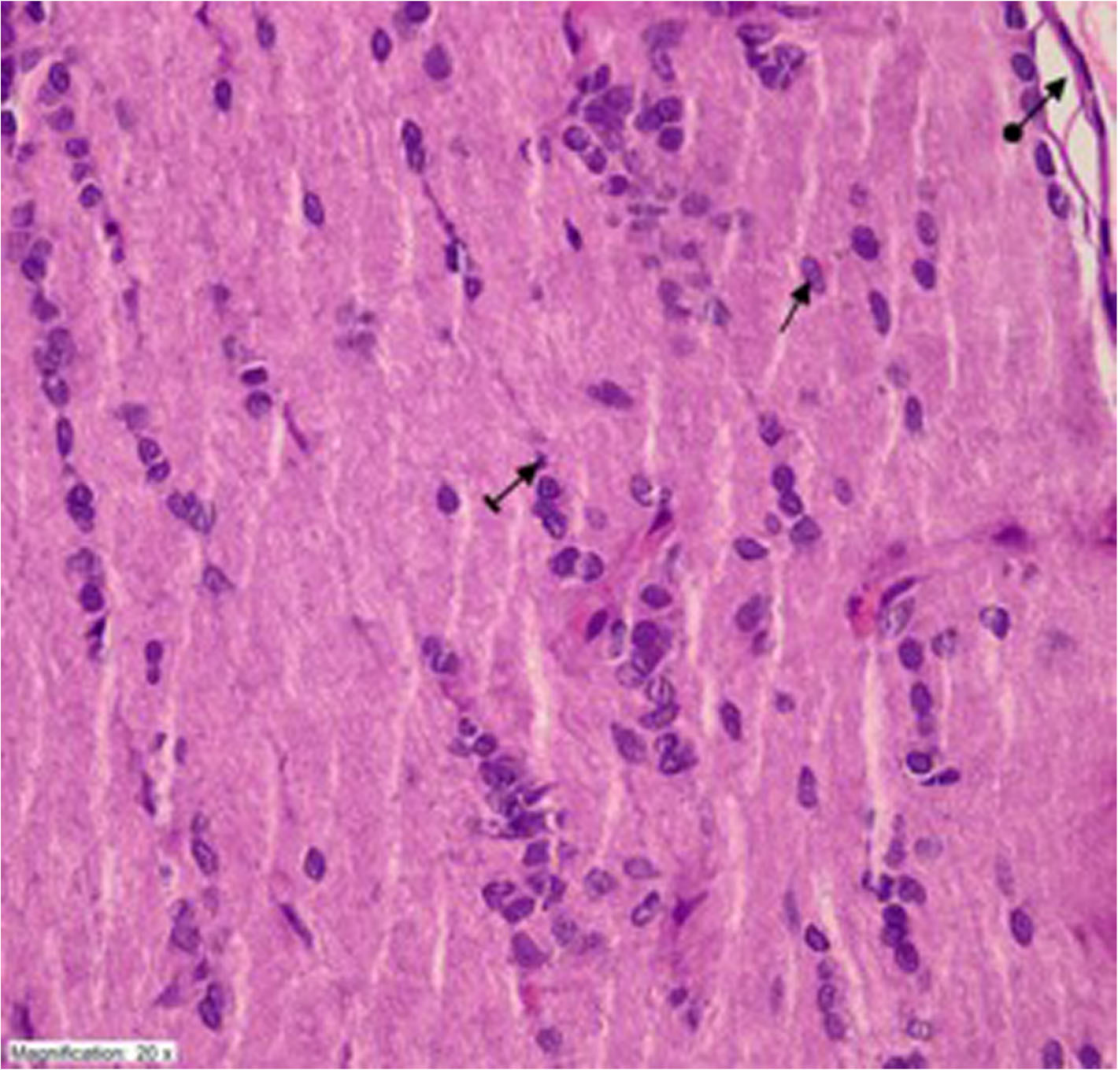
The appearance of optic nerve tissue was similar with the healthy controls in TPG group with healthy nerve sheet (round arrow), healthy astrocyte (straight arrow), and healthy oligodendrocyte (striped arrow) (HEX400)

The histopathological findings are shown in Figs. 1, 2, 3 and 4. Regarding these findings, in EtOH group there was obvious destruction and edema with hemorrhage and dilated blood vessels in optic nerve. On the other hand, the histopathological findings in TEt-20 group were near normal except a mild edema.

## Discussion

In this study we investigated the effects of thiamine pyrophosphate on protection from the destructive effects of chronic ethanol administration on eye tissue in an experimental model and we have determined that: 1) Six weeks of ethanol administration was associated with an increased systemic oxidative damage and augmented destruction on eye tissue; 2) concomitant administration of thiamine pyrophosphate was significantly effective on protection from this damage at both systemic and ocular planes.

Chronic alcohol consumption is clearly known to enhance the production of reactive oxygen species and augment the oxidative stress and oxidative stress is one of the main mechanisms of ethanol induced tissue damage [18, 19]. Expression of Cytochrome p450 2E1 (CYP2E1) mRNA, CYP2E1 protein activity, and production of reactive oxygen species were determined to be induced by ethanol in a concentration-dependent manner in retinal pigment epithelium [20]. Moreover, chronic alcohol consumption was also associated with the ultra-structural alterations on cornea [21].

Reactive oxygen species produced as a result of ethanol consumption reacts with biological macromolecules such as DNA and results in the lipid peroxidation. MDA is the end product of lipid peroxidation and regarded as an indicator of measure of oxidative stress [22]. Decreased serum and tissue MDA levels determined in our study was also showing the success of thiamine pyrophosphate in preventing oxidative stress development. Glutathione is an important non-enzymatic antioxidant decreasing lipid peroxidation in tissues [23]. We have determined significantly higher reduced glutathione levels in serum and tissues in thiamine pyrophosphate group compared with the ethanol group showing the anti-oxidant effects of thiamine pyrophosphate. We also investigated the IL-1β and TNF-α level which are the cytokines that stimulate cells to intensify the inflammatory response [24] and we have determined that those inflammatory markers were significantly increased with ethanol consumption and this increase was also prevented by concomitant thiamine pyrophosphate administration.

In previous literature there are only a few studies investigating the role of antioxidant agents in prevention of ethanol-induced ocular abnormalities. Johnsen-Soriano et al. [25] reported that chronic alcohol consumption was causing an alteration on the retinal redox status which was returned with a biologically active selenium-organic compound treatment. Parnell et al. [26] reported that N-acetylcysteine was effective to reduce the ocular teratogenic effects of ethanol in an experimental model. For the first time in literature we have determined that; thiamine pyrophosphate was effective in prevention of oxidative stress development and optic neuropathy induced by ethanol.

Thiamine is a water-soluble vitamin having a critical role in glucose metabolism. Classically, thiamine deficiency results in an acute confusional state and ataxia, while ophthalmoparesis and optic disc swelling may also take place rarely [27, 28]. The role of thiamine pyrophosphate treatment has been studied in some ocular diseases before. Cinici et al. [29] reported that thiamine pyrophosphate significantly reduced the degree of hyperglycemia-induced retinopathy by preventing the oxidative damage. In another recent study, thiamine pyrophosphate but not the thiamine was determined as having protective effects on retinal tissues from ethambutol-induced oxidative damage [11]. The latter one was an important study since it was comparing thiamine and the thiamine pyrophosphate; and its results can be interpreted as ‘administration of thiamine pyrophosphate might lead to a slower, but longer lasting thiamine accumulation in the blood than thiamine administration’. However, this hypothesis should be checked by measuring serum thiamine levels at different time intervals. These recent studies were also supporting our findings that thiamine pyrophosphate was having protective effects on oxidative stress induced ocular damage.

Of course this study has some limitations. First is that; this is an experimental study and direct dosage and pharmacokinetic comparisons between rats and humans may be difficult due to the inter-species differences in metabolism and administration paradigms. Second is the low number of rats that is especially kept low regarding the animal rights. We also did not measure the serum or tissue thiamine concentrations at different time periods, which is another limitation of this study. Determination of serum or tissue thiamine concentrations would elucidate the effectiveness of administered thiamine pyrophosphate.

## Conclusions

In conclusion in this study we have determined that, thiamine pyrophosphate administration was effective in prevention of oxidative stress and inflammation induced by chronic ethanol consumption and for the first time in literature we have determined that at histopathological level, thiamine pyrophosphate was protective for the ethanol induced optic neuropathy. There was an apparent destruction in ethanol administered group in histopathological analyses with an augmented oxidative stress and all those alterations were prevented with concomitant thiamine pyrophosphate administration. In future studies, investigating the effects at different time intervals after starting ethanol administration and/or sacrificing the rats at different time points to see the time-bound beneficial effects of thiamine pyrophosphate and reversibility of the optic nerve damage on histopathology would be highly beneficial. Moreover, gene based analysis may also show the pathophysiological aspect of these effects of thiamine pyrophosphate. Clinical studies are also warranted to define the role of thiamine pyrophosphate in prevention of other ocular diseases induced by oxidative stress and inflammation.

## Data Availability

The datasets used and/or analyzed during the current study are available from the corresponding author on reasonable request.

## Abbreviations

GSH: Reduced glutathione
IL-1β: Interleukin 1 beta
MDA: Malondialdehyde
TNF-α: Tumor necrosis factor alpha
